# An Investigation on the Wear Resistance and Fatigue Behaviour of Ti-6Al-4V Notched Members Coated with Hydroxyapatite Coatings

**DOI:** 10.3390/ma9020111

**Published:** 2016-02-16

**Authors:** Reza H Oskouei, Khosro Fallahnezhad, Sushmitha Kuppusami

**Affiliations:** 1 Medical Device Research Institute, School of Computer Science, Engineering and Mathematics, Flinders University, Tonsley 5042, Australia; 2 Discipline of Mechanical Engineering, School of Computer Science, Engineering and Mathematics, Flinders University, Tonsley 5042, Australia; khosro.fallahnezhad@flinders.edu.au (K.F.); kupp0002@uni.flinders.edu.au (S.K.)

**Keywords:** titanium alloys, hydroxyapatite coatings, wear, fatigue

## Abstract

In this study, surface properties of Ti-6Al-4V alloy coated with hydroxyapatite coatings were investigated. Wear resistance and fatigue behaviour of samples with coating thicknesses of 10 and 50 µm as well as uncoated samples were examined. Wear experiments demonstrated that the friction factor of the uncoated titanium decreased from 0.31 to 0.06, through a fluctuating trend, after 50 cycles of wear tests. However, the friction factor of both the coated samples (10 and 50 µm) gradually decreased from 0.20 to 0.12 after 50 cycles. At the end of the 50th cycle, the penetration depth of the 10 and 50 µm coated samples were 7.69 and 6.06 µm, respectively. Fatigue tests showed that hydroxyapatite coatings could improve fatigue life of a notched Ti-6Al-4V member in both low and high cycle fatigue zones. It was understood, from fractography of the fracture surfaces, that the fatigue zone of the uncoated specimens was generally smaller in comparison with that of the coated specimens. No significant difference was observed between the fatigue life of coated specimens with 10 and 50 µm thicknesses.

## 1. Introduction

Owing to their excellent mechanical properties and biocompatibility, metallic biomaterials are largely used in different medical applications such as orthopaedic implants, bone plates, dental implants and cardiovascular devices [1,2,3]. It is reported that 70%–80% of implants used for biomedical applications are made of metals and metallic alloys [4] for which high strength, low elastic modulus, excellent wear and corrosion resistance, and good biocompatibility are of paramount importance [5]. Due to the exposure to the corrosive environment of the body, metallic implants may suffer from corrosion which can be intensified by the effect of wear induced by coming into contact with a hard surface [6]. This can result in toxicity, adverse local tissue reaction and ultimately failure of the metallic implant [7,8]. Given the cyclic nature of the mechanical loads applied during physical activities, fatigue behaviour of implants is also important.

Surface modification methods, particularly coatings, have been extensively used to improve mechanical and surface properties of metals in different applications [9,10,11,12]. Wang and Zreiqat [3] reviewed a wide range of surface coatings used for metallic biomaterials along with their deposition methods and properties including corrosion, wear, biocompatibility and antibacterial activity. Hydroxyapatite (HA) coatings [Ca_10_(PO_4_)_6_(OH)_2_] are widely used in prosthetic applications as they possess a similar chemical composition and crystal unit to apatite of the bone [13,14]. It favourably accelerates the bone formation process as it contains calcium phosphate [15] which makes it suitable for bone substitution and reconstruction [10,15,16,17].

HA coatings have shown success in implant fixation (*in-vivo*) which enhances the implant life by increasing the strength and rate of implant integration [18]. The dense layer of coating onto the surface of implant material is mainly for bio-integration and increased implant stability to the bone tissue [19,20]. In addition to its bioactive role, HA coatings can minimise ion release of metals in the physiological environment of the body [15].

However, HA coatings have a brittle nature and low strength under wear conditions [21,22]. Balani *et al*. [23] evaluated wear behaviour of hydroxyapatite coatings reinforced by plasma-sprayed carbon nanotubes in a simulated body fluid environment. Their results demonstrated considerable improvements in the fracture toughness (56%) and crystallinity (27%). Moreover, coated implants may suffer from fatigue damage under cyclic loading in the body. Thus, fatigue response of coated metallic biomaterials needs to be investigated. The effect of surface coatings on the fatigue behaviour of biomaterials, especially metals, has been investigated previously. Apachitei *et al.* [24] investigated fatigue behaviour of Ti-6Al-4V and Ti-6Al-7Nb alloys coated with plasma electrolytic oxidation coatings. Their study showed that this type of coatings reduces fatigue resistance of these alloys. Vadiraj and Kamaraj [25] studied the effect of titanium nitride coatings on the fretting wear properties of titanium alloys. They concluded that physical vapour deposition (PVD) TiN coatings can improve fretting fatigue properties of titanium alloys.

There have been however limited studies on the fatigue behaviour of metallic biomaterials coated with HA coatings. Lynn *et al.* [26] investigated the effect of HA coating thickness on the fatigue behaviour of Ti-6Al-4V. They found that changing the thickness of atmospheric plasma-spray (APS) HA coatings from 0 to 100 µm does not affect the fatigue life of the alloy, but thickness of 150 µm considerably reduces the fatigue resistance of Ti-6Al-4V. They also investigated the effect of heat treatment on the fatigue behaviour of this alloy coated with HA coatings [27]. It was shown that a heat treatment at 400 °C for 90 h significantly reduces the fatigue resistance of this alloy. Fatigue tests in these studies were limited to one constant stress amplitude (620 MPa); whereas, stress *versus* number of cycles (S-N) curves could be more useful in understanding the overall fatigue behaviour. Also, implants usually have complex geometries with fillets and reduced sections which can act as a notch. Hence, fatigue behaviour of a notched implant material coated with HA coatings should be studied.

In this study, surface properties of hydroxyapatite coatings deposited onto Ti-6Al-4V substrate is characterised. Two thickness of 10 and 50 µm were studied. Wear tests were conducted to evaluate wear resistance of the HA coating layer with two different thicknesses. The effect of HA coatings thickness on the fatigue behaviour of Ti-6Al-4V notched specimens was also investigated in different cyclic load levels. Fatigue crack initiations and propagations were examined by scanning electron microscopy (SEM) to identify crack origins and detect coating delaminations after failure.

## 2. Experimental Section

### 2.1. Sample Preparation

Titanium alloys are one of the most commonly used metals for biomedical applications because they offer superior properties which include low density, moderate elastic modulus (110 GPa), good corrosion resistance and high strength [2]. In this work, Ti-6Al-4V alloy, ELI (Extra Low Interstitials) grade (Grade 23) was used as substrate. Ti-6Al-4V ELI contains reduced amounts of oxygen, nitrogen, carbon and iron. In this alloy, lower interstitials can improve ductility and offer better fracture toughness. The alloy was purchased as a 0.50-inch-diameter bar from Magellan Metals, South Norwalk, CT, USA. Small disc samples were cut from the bar using Secotom-50 (Struers, Ballerup, Denmark). The samples had the same diameter as the bar (12.7 mm) and a 5-mm-thickness. After the cutting process, one face of the discs was mirror polished using Tegramin-25 (Struers, Ballerup, Denmark) to remove surface scratches.

**Figure 1:**
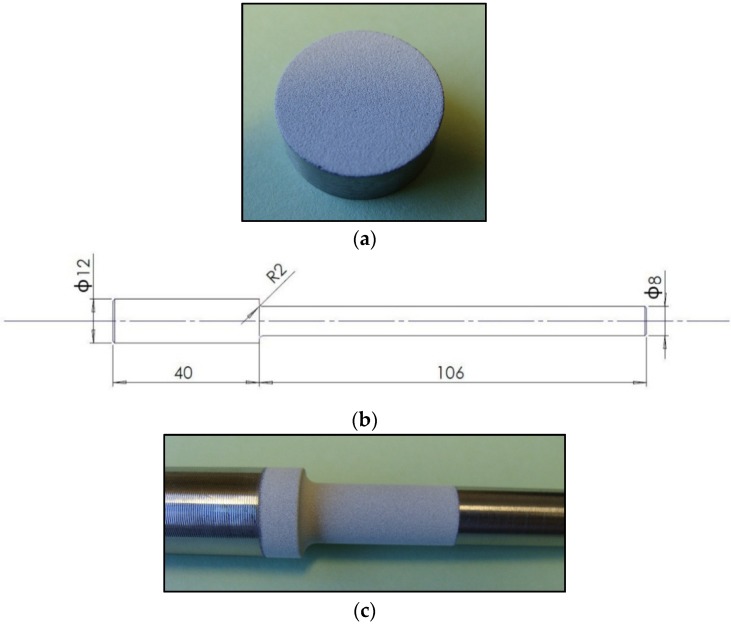
(**a**) Ti-6Al-4V disc sample coated with hydroxyapatite coatings; (**b**) fatigue test specimen, dimensions in mm; and (**c**) notched section of the fatigue test specimen coated with hydroxyapatite coatings.

Three batches of samples were prepared. These included uncoated titanium samples, HA coated samples with a coating thickness of 5–15 µm, and HA coated samples with a coating thickness of 45–55 µm. Hydroxyapatite coatings were deposited onto the titanium surface using a thermal plasma spray process by Himed, Old Bethpage, NY, USA. All the samples were coated in the targeted thickness ranges. Nominal thicknesses of 10 and 50 µm were used in this study for the afore-mentioned thickness ranges, respectively. Prior to applying the HA coating, the disc samples were degreased using 3% Alconox, distilled water and 99% alcohol. The polished surface was then grit-blasted with apatitic abrasive (MCD, 180–300 µm, Himed, Old Bethpage, NY, USA) and passivated as per ASTM F86-04 [28]. The coated sample is shown in Figure 1a. Fatigue test specimens with a reduced section in diameter, as shown in Figure 1b, were manufactured from the Ti-6Al-4V bar. Similar to the disc samples, fatigue test specimens were coated with hydroxyapatite coatings with two thicknesses of 10 and 50 µm. The notched section was only coated where final fracture was expected to occur (Figure 1c).

### 2.2. Coating Characterisations

The coated disc samples were used to characterise surface properties of the coating. Scanning electron microscopy, SEM, (FEI, Inspect F50, Hillsboro, OR, USA) and energy dispersive spectroscopy (EDS) were used to examine the microstructure and chemical composition of the coating and Ti-6Al-4V substrate. In addition, surface topography and roughness of the coating and titanium substrate were characterised using a surface profilometer (WYKO NT9100, Veeco, Plainview, NY, USA) to determine the average roughness parameters of the surface.

### 2.3. Wear Assessments

Wear resistance of the HA coated samples and uncoated titanium sample was examined using a scratch tester mounted on an IBIS nano-indentation system (M/S Fisher-Cripps Laboratories Pvt. Limited, Killarney Heights, NSW, Australia). This technique was used to evaluate wear resistance of the material by applying a certain normal force to the surface along with a displacement amplitude for a number of passes. A 200 µm diamond sphero-cone tip was used with a normal force of 50 mN and a displacement amplitude of 100 µm (in both left and right directions) with a velocity of 5 µm/s over the surface. The coefficient of friction and penetration depth were measured and recorded during the test. The wear test was performed for 50 passes for each sample and the data were recorded in every two passes.

### 2.4. Fatigue Tests

**Figure 2:**
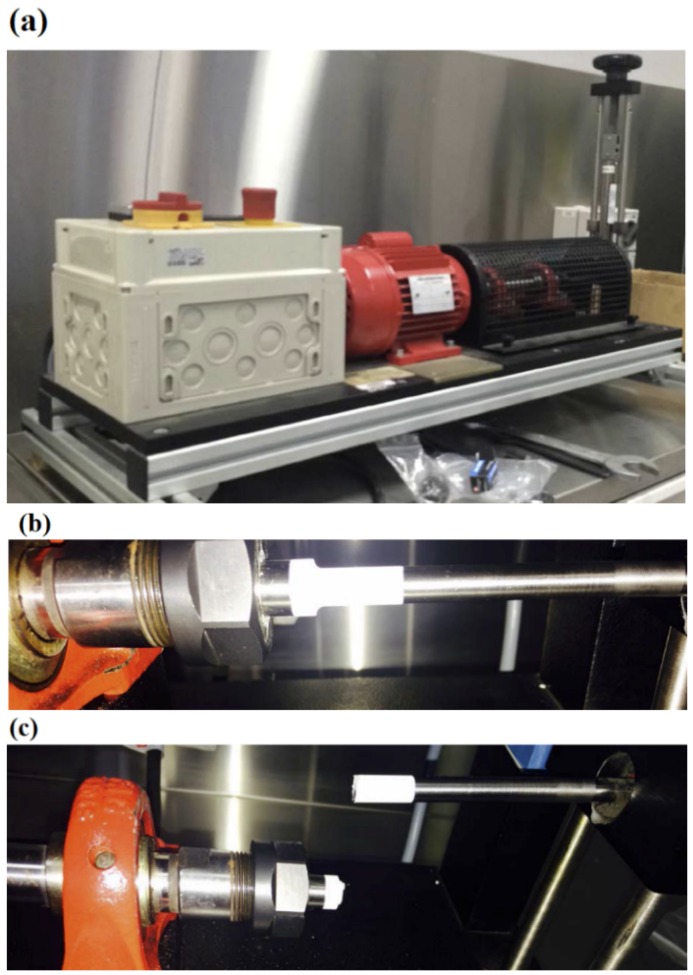
(**a**) Fatigue testing machine, GUNT WP 140; (**b**) HA coated specimen under fatigue loading; and (**c**) HA coated specimen after fatigue failure.

Fatigue tests were conducted to study the effect of HA coatings on the fatigue resistance of Ti-6Al-4V alloy with a notch, as shown in Figure 1b. To accomplish this, a rotating fatigue testing machine (G.U.N.T. Gerätebau GmbH, Hamburg, Germany, model WP 140) was used to apply completely reversed bending stresses (stress ratio of *R* = −1) with a frequency of 46.7 Hz (spindle speed of 2800 rpm), as shown in Figure 2a. Using a calibrated spring balance together with a floating bearing, the specimens were loaded to desired levels. Depending on the load level, bending stresses applied to the top and bottom faces of the specimens were between 450 and 600 MPa. This range of stress amplitudes was found to be reasonable to address both low cycle and high cycle fatigue behaviours for the specimens. Fatigue life was considered as the complete separation of the specimens (Figure 2c). For each type of specimen and stress amplitude, three fatigue tests were performed and the average fatigue life was used in S-N curves.

## 3. Results and Discussion

### 3.1. Coating Characteristics

**Figure 3:**
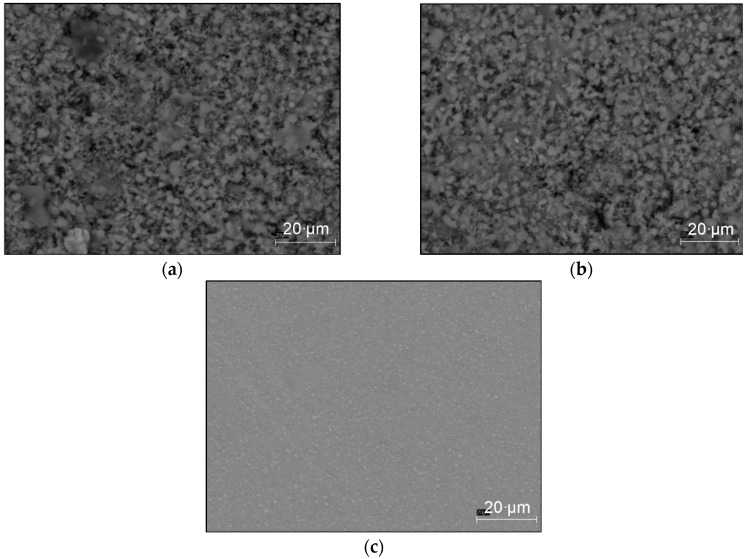
SEM image of: (**a**) HA coated sample with thickness of 10 µm; (**b**) HA coated sample with thickness of 50 µm; and (**c**) uncoated Ti-6Al-4V sample.

SEM images of both the HA coated samples (with 10 and 50 µm thicknesses) and the uncoated titanium sample are shown in Figure 3. The image of both thicknesses demonstrate that the hydroxyapatite coatings are deposited uniformly onto the titanium substrate surface. It is apparent that in spite of some micro-voids, coatings are tightly adhered together. The mirror polished surface of the uncoated Ti-6Al-4V can be seen in Figure 3c before applying the HA coatings.

**Figure 4:**
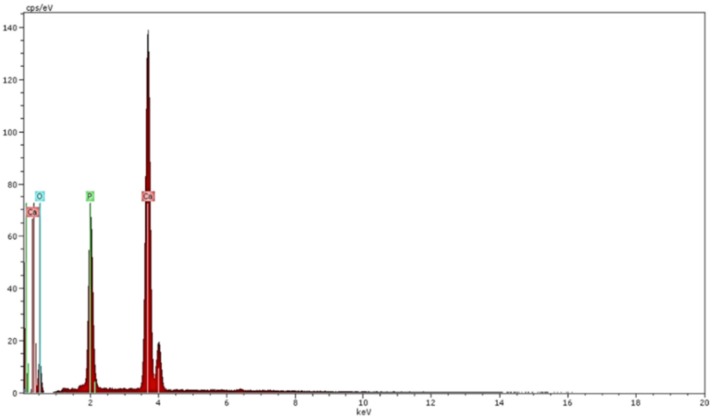
EDS results of hydroxyapatite coatings used in this study.

EDS results of the coating layer are given in Figure 4. It is noted that the EDS results were the same for both thicknesses of the coating layer, as expected. The elemental analysis confirmed the presence of calcium (55%), phosphorus (20%) and oxygen (25%), all in percentage weight (wt%), in hydroxyapatite coatings used in this work.

**Figure 5:**
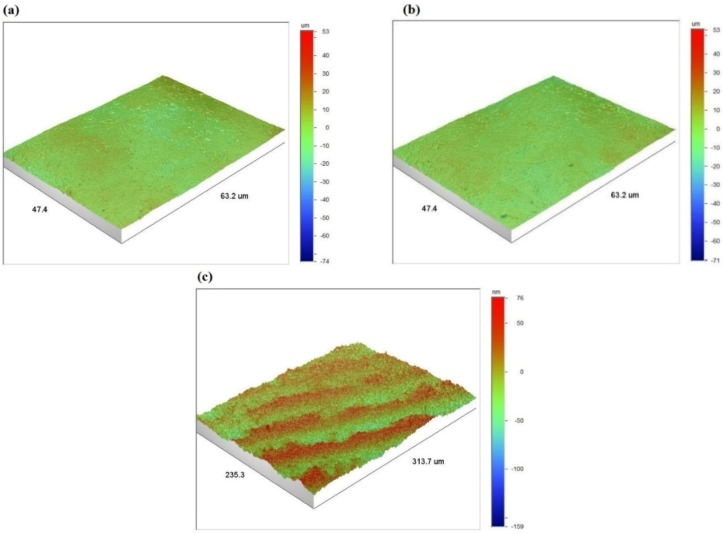
3D surface profile of: (**a**) HA coated sample with thickness of 10 µm; (**b**) HA coated sample with thickness of 50 µm; and (**c**) uncoated Ti-6Al-4V sample.

The surface roughness parameters for uncoated, 10 µm coated and 50 µm coated samples were recorded in five different areas over the sample surface. The arithmetic average (*R_a_*), root mean squared (*R_q_*) and maximum vertical distance between the highest and lowest data points (*R_max_*) were determined, as given in Table 1. As can be seen, there is no considerable difference in the surface roughness measures of the two coated samples indicating that the roughness of the 10 and 50 µm coated samples is very similar. This confirms that the surface roughness, as a component of the surface texture, was independent of the coating thickness. However, the surface roughness of the uncoated titanium sample was much smaller (*R_a_* = 15.49 nm against *R_a_* = 2.60–2.66 µm) which was obviously because of the highly polished surface finish of the substrate. The 3D surface profile for one of the five scanned areas of these samples is shown in Figure 5 where the similarity between the two coated samples can be seen in terms of the surface profile.

**Table 1:** Surface roughness measures (*R_a_*, *R_q_* and *R_max_*) of different areas for three batches of samples.

Tested Areas	10 µm Coated Sample (µm)	50 µm Coated Sample (µm)	Uncoated Sample (nm)
**Area 1**	*R_a_* = 2.46 R_q_ = 3.15 R_max_ = 12.4	*R_a_* = 2.60 R_q_ = 3.19 R_max_ = 13.00	*R_a_* = 19.18 R_q_ = 23.39 R_max_ = 10.10
**Area 2**	*R_a_* = 2.47 R_q_ = 3.16 R_max_ = 12.20	*R_a_* = 3.14 R_q_ = 3.84 R_max_ = 13.00	*R_a_* = 13.99 R_q_ = 17.79 R_max_ = 9.20
**Area 3**	*R_a_* = 2.90 R_q_ = 3.68 R_max_ = 14.30	*R_a_* = 2.42 R_q_ = 3.04 R_max_ = 11.62	*R_a_* = 16.82 R_q_ = 22.15 R_max_ = 8.76
**Area 4**	*R_a_* = 2.21 R_q_ = 2.90 R_max_ = 13.50	*R_a_* = 2.67 R_q_ = 3.40 R_max_ = 12.80	*R_a_* = 13.11 R_q_ = 16.61 R_max_ = 8.72
**Area 5**	*R_a_* = 2.97 R_q_ = 3.78 R_max_ = 13.90	*R_a_* = 2.47 R_q_ = 3.02 R_max_ = 11.00	*R_a_* = 14.35 R_q_ = 18.13 R_max_ = 9.14
**Average**	*R_a_* = 2.60 R_q_ = 3.33 R_max_ = 13.26	*R_a_* = 2.66 R_q_ = 3.30 R_max_ = 12.28	*R_a_* = 15.49 R_q_ = 19.61 R_max_ = 9.18

### 3.2. Wear Resistance Results

**Figure 6:**
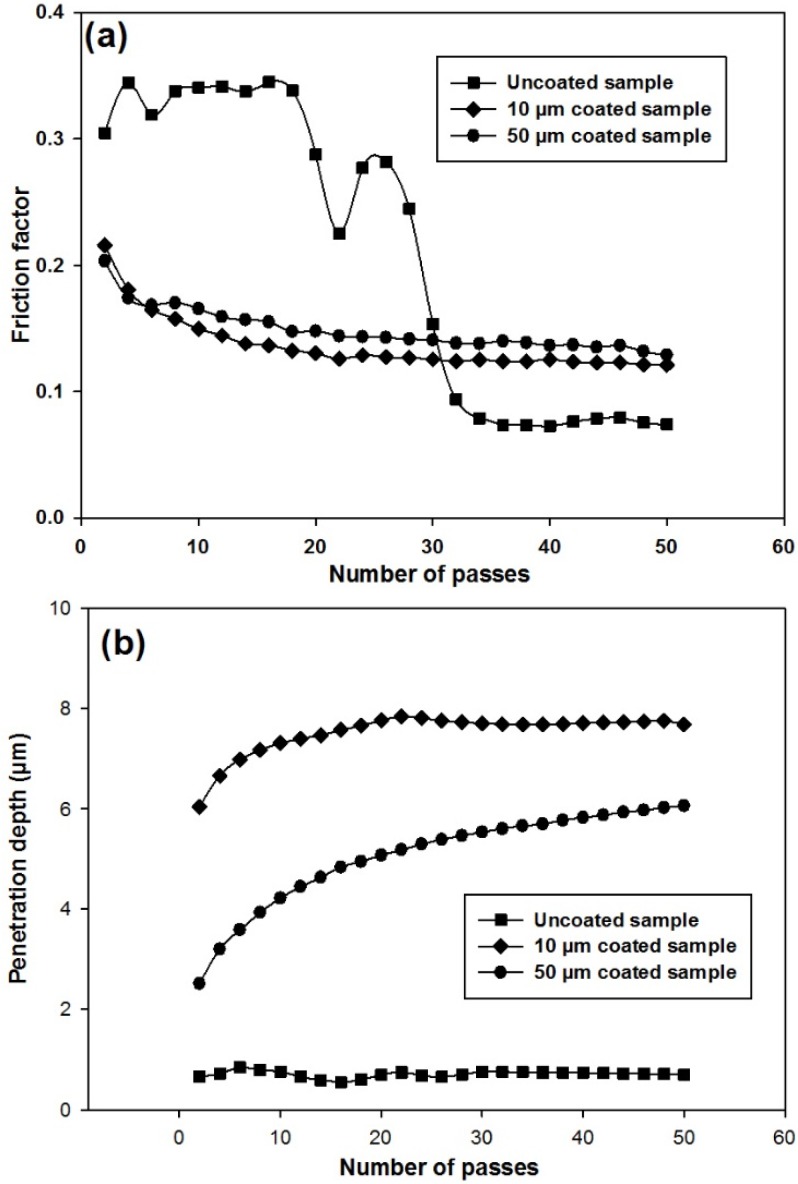
Wear test results of: (**a**) friction factor *versus* number of passes; (**b**) depth of penetration versus number of passes for three batches of samples.

Friction behaviour of the three samples is shown in Figure 6a in the form of friction factor through 50 passes of the sliding test. It is apparent that the friction factor of the uncoated titanium sample was considerably greater than that of the coated samples with both thicknesses at the beginning of the test. The friction factor was approximately steady by around 18 passes after which, there was an overall reduction in the friction factor of the uncoated sample. The trend remained constant again from the 34th to the 50th pass of the test. Variations in friction factor were more uniform for both the coated samples. At the start of the test, the friction factor for these two samples was around 0.20. With an increase in the number of passes, the friction factor gradually decreased to 0.12. The interesting point is that at the beginning of the test, the uncoated sample had a greater friction factor in comparison with the coated samples. However, after 50 passes, the friction factor of the uncoated sample was found to be smaller than the coated samples. Such behaviours indicate that friction factor varies with the number of passes of the sliding test. Figure 6b shows the behaviour of the penetration depth *versus* the number of passes from the wear experiments on the three samples. It can be seen from the plots that the penetration depth for the uncoated sample was almost constant (0.74 µm) during the wear test; whereas, the penetration depth of the coated samples gradually increased during the test. The penetration depth of the 10 µm coating was greater than that of the 50 µm coating. The maximum penetration depths for the 10 and 50 µm coated samples were 7.69 and 6.06 µm, respectively. Given the maximum penetration depths were still within the thickness range of both the coated samples (10 and 50 µm), the friction factors in both the coated samples, as shown in Figure 6a, were found to be similar during the wear experiments.

### 3.3. Fatigue Test Results

**Figure 7:**
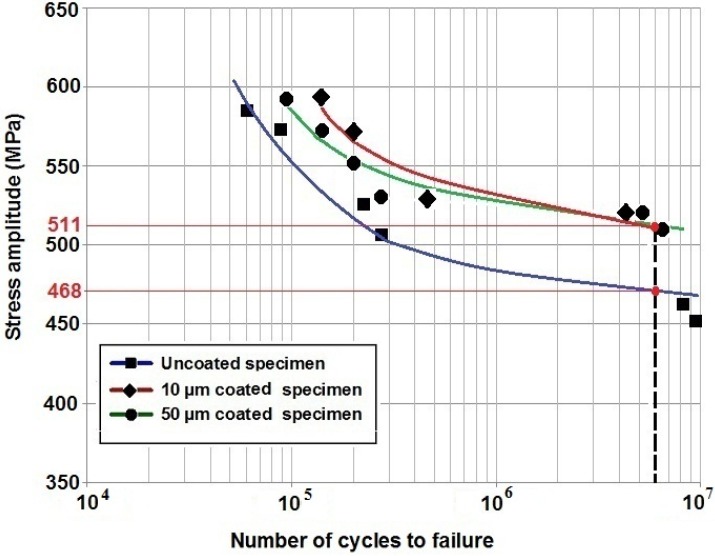
S-N curves of uncoated Ti-6Al-4V, 10 µm HA coated and 50 µm HA coated specimens, *R* = −1.

Figure 7 presents the S-N curves of the three batches of tested specimens. In general, HA coatings were found to improve the fatigue resistance of Ti-6Al-4V alloy, especially at high cycle fatigue zone. It can be seen that under stress amplitudes between 520 and 590 MPa, 10 µm coated specimen experienced the highest number of cycles before failure. For instance, under 590 MPa, the 10 and 50 µm coated samples failed after 139,000 and 96,000 cycles, respectively. However, the uncoated specimen had 58,000 cycles. At 6 million cycles, two red points are marked on the curves for the uncoated and coated specimens. These two points were considered as the fatigue limit that were 468 and 511 MPa for the uncoated and both coated specimens, respectively.

**Figure 8:**
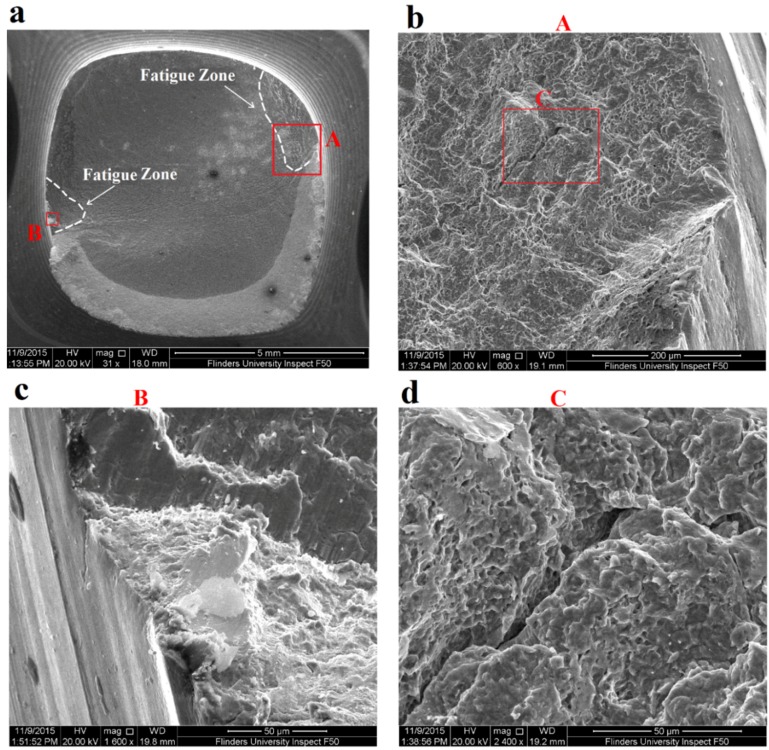
SEM images of fatigue fracture surface of uncoated Ti-6Al-4V specimen failed under stress amplitude of 570 MPa and *R* = −1: (**a**) fracture surface showing fatigue zones; (**b**) fatigue crack initiation site A; (**c**) fatigue crack initiation site B; and (**d**) higher magnification image of a deep crack in area C.

**Figure 9:**
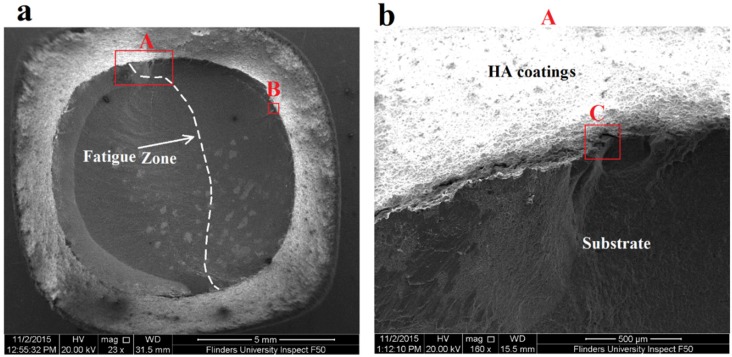

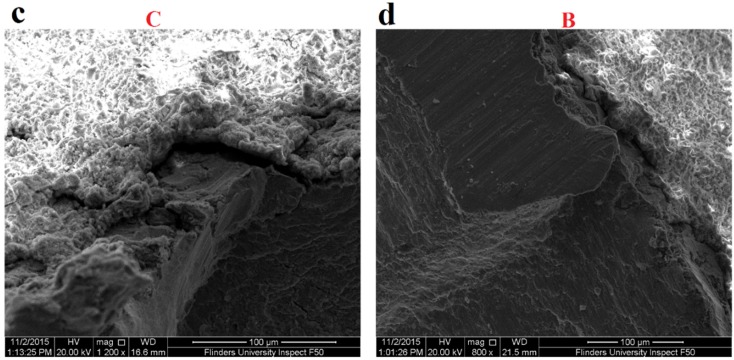
SEM images of fatigue fracture surface of 10 µm HA coated specimen failed under stress amplitude of 570 MPa and *R* = −1: (**a**) fracture surface showing fatigue zone; (**b**) fatigue crack initiation site A; (**c**) higher magnification image of fatigue crack initiation point C; and (**d**) higher magnification image of fatigue crack initiation site B.

**Figure 10:**
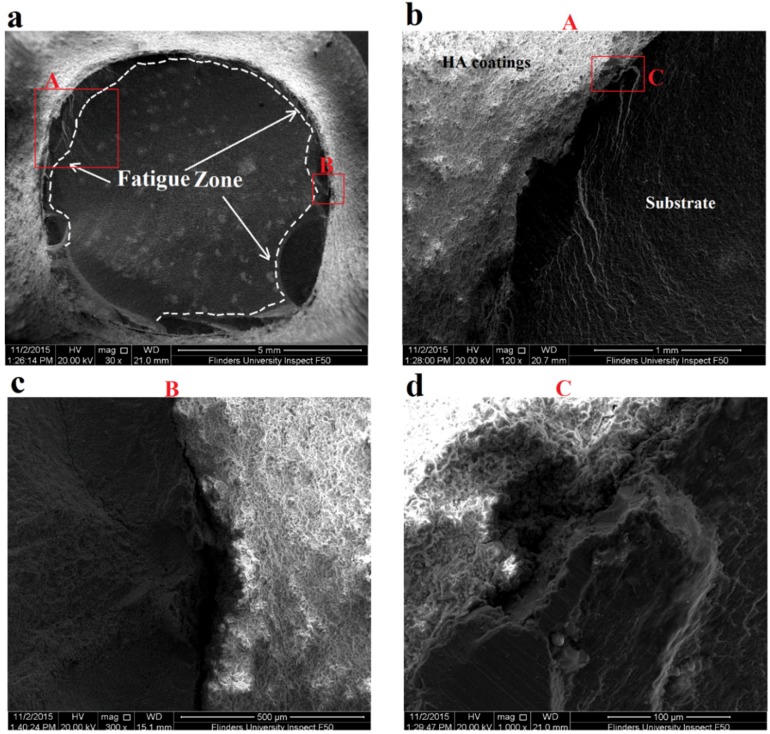
SEM images of fatigue fracture surface of 50 µm HA coated specimen failed under stress amplitude of 570 MPa and *R* = −1: (**a**) fracture surface showing fatigue zones; (**b**) fatigue crack initiation site A; (**c**) fatigue crack initiation site B; and (**d**) higher magnification image of fatigue crack initiation point C.

Fatigue fracture surfaces of 10 and 50 µm coated specimens and uncoated specimen were investigated using SEM to evaluate fatigue crack initiations and propagations along with their fatigue zone over the fracture surface. Furthermore, it was important to evaluate the coating adherence to the substrate after fatigue failure. SEM images of three specimens including uncoated titanium specimen, 10 µm HA coated and 50 µm HA coated specimens failed at stress amplitude of 570 MPa are shown in Figure 8, Figure 9 and Figure 10, respectively. The figures present a low magnification image of the whole fracture surface in which fatigue zones are indicated. There are also higher magnification images showing fatigue crack initiation sites and crack propagations towards the specimen centre. Due to the presence of the notch, and as a consequence, the stress concentration all around the specimen circumference, cracks initiated from the edge of the notch, as expected. The interesting finding was that both the 10 and 50 µm coated specimens demonstrated the presence of the coating layer at the edge of the fracture surface which confirms a good adherence between the HA coating and substrate under cyclic loading (Figure 9b and Figure 10b).

**Figure 11:**
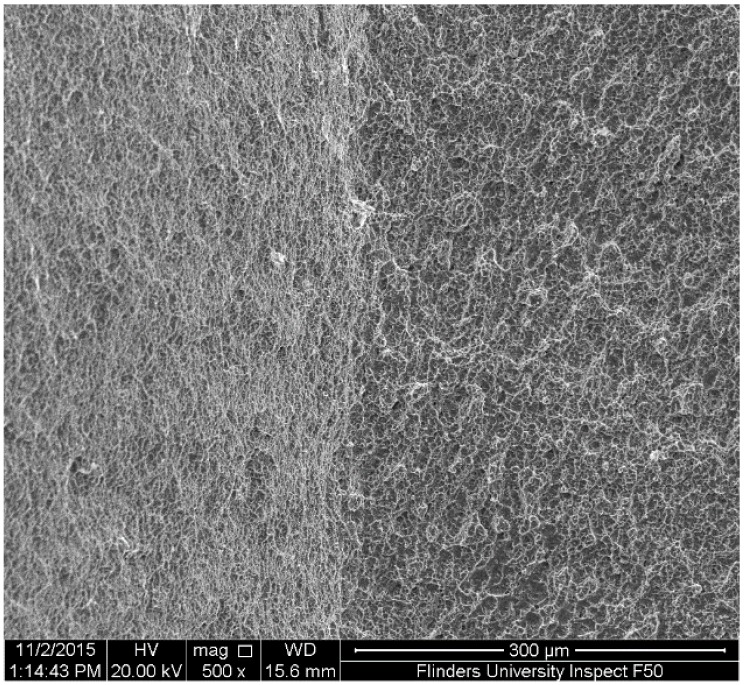
SEM image of the final fracture zone, taken from the 10 µm HA coated specimen.

An investigation of the SEM images also showed that the fatigue zone of the uncoated titanium specimen was generally smaller in comparison with the coated specimens. It can be concluded that the uncoated specimen had less number of cycles for the crack propagation stage and thus smaller fatigue zones were formed over the fracture surface. In other words, under the same stress amplitude, coated specimens tolerated more cycles for the crack propagation stage resulting in an improvement in the fatigue life, as can be seen also in the S-N curves of Figure 7. Moreover, the fatigue experiments showed no significant differences between the 10 and 50 µm coated specimens. The SEM images of these coated specimens showed similar fatigue fracture features mainly in terms of the presence of multiple crack initiations and amount of area that is influenced by crack propagations. This together with the fact that all the crack initiations occurred at the interface of the titanium substrate and the coating layer could explain the similar fatigue behaviour of both the 10 and 50 µm coated specimens. Final fracture zone in all the three specimens showed ductile fracture features. Figure 11 demonstrates the final fracture zone of the 10 µm coated specimen in which dimples can be seen over the surface.

## 4. Conclusions

Surface properties of Ti-6Al-4V substrate coated with hydroxyapatite coatings were characterised. SEM images of the coated samples demonstrated that HA coatings were uniformly deposited onto the substrate surface. The surface roughness measures (*R_a_, R_q_ and R_max_*) for coated samples with 10 and 50 µm thicknesses were found to be similar. However, roughness parameters were much smaller in the mirror-polished uncoated titanium sample. The friction factor of both the coated samples was also similar decreasing from 0.20 to 0.12 after 50 cycles of the sliding test. This similarity was because the maximum penetration depths of the wear experiments were well within the thickness ranges of the coating. Although at the start of the test the friction factor of the uncoated sample was greater than the coated samples, after 50 cycles, the friction factor became less than that of the coated samples. Fatigue resistance of a notched specimen of Ti-6Al-4V coated with hydroxyapatite coatings was also studied. S-N curves of the coated and uncoated specimens were presented. It was found that HA coatings could improve fatigue life of the titanium notched specimen in both low and high cyclic fatigue zones. SEM images of the fracture surface showed smaller fatigue zones in the uncoated specimen in comparison with the coated ones. The coating layer was found to remain adhered to the substrate under fatigue loading. Moreover, the fatigue behaviour of both the 10 and 50 µm coated specimens was found to be similar. This was justified based on the similarity between the fatigue fracture features that was observed in both the coated specimens.
